# Role of Overexpressed Transcription Factor FOXO1 in Fatal Cardiovascular Septal Defects in Patau Syndrome: Molecular and Therapeutic Strategies

**DOI:** 10.3390/ijms19113547

**Published:** 2018-11-10

**Authors:** Adel Abuzenadah, Saad Alsaedi, Sajjad Karim, Mohammed Al-Qahtani

**Affiliations:** 1 Center of Excellence in Genomic Medicine Research, Faculty of Applied Medical Sciences, King Abdulaziz University, P.O. Box 80216, Jeddah 21589, Saudi Arabia; aabuzenadah@kau.edu.sa (A.A.); mhalqahtani@kau.edu.sa (M.A.-Q.); 2 King Fahd Medical Research Center, King Abdulaziz University, P.O. Box 80216, Jeddah 21589, Saudi Arabia; 3 Department of Pediatric, Faculty of Medicine, King Abdulaziz University Hospital, King Abdulaziz University, P.O. Box 80215, Jeddah 21589, Saudi Arabia; salsaedi@hotmail.com

**Keywords:** Patau Syndrome, cytogenetics, FOXO1, transcription factor, molecular pathways, bioinformatics, molecular docking, and drug design

## Abstract

Patau Syndrome (PS), characterized as a lethal disease, allows less than 15% survival over the first year of life. Most deaths owe to brain and heart disorders, more so due to septal defects because of altered gene regulations. We ascertained the cytogenetic basis of PS first, followed by molecular analysis and docking studies. Thirty-seven PS cases were referred from the Department of Pediatrics, King Abdulaziz University Hospital to the Center of Excellence in Genomic Medicine Research, Jeddah during 2008 to 2018. Cytogenetic analyses were performed by standard G-band method and trisomy13 were found in all the PS cases. Studies have suggested that genes of chromosome 13 and other chromosomes are associated with PS. We, therefore, did molecular pathway analysis, gene interaction, and ontology studies to identify their associations. Genomic analysis revealed important chr13 genes such as FOXO1, Col4A1, HMGBB1, FLT1, EFNB2, EDNRB, GAS6, TNFSF1, STARD13, TRPC4, TUBA3C, and TUBA3D, and their regulatory partners on other chromosomes associated with cardiovascular disorders, atrial and ventricular septal defects. There is strong indication of involving FOXO1 (Forkhead Box O1) gene—a strong transcription factor present on chr13, interacting with many septal defects link genes. The study was extended using molecular docking to find a potential drug lead for overexpressed FOXO1 inhibition. The phenothiazine and trifluoperazine showed efficiency to inhibit overexpressed FOXO1 protein, and could be potential drugs for PS/trisomy13 after validation.

## 1. Introduction

Patau Syndrome (PS) is a rare congenital anomaly due to the presence of an extra chromosome 13 popularly called trisomy 13 [1]. In spite of being the least common, it is the severest of all autosomal trisomies indicated by a prevalence rate of 1:5000 to 1:20,000 [2,3]. The syndrome is associated with a host of congenital anomalies including central nervous system (CNS) defects, midline abnormalities, eye and ear anomalies, cardiac defects, apnea, orofacial flaws, gastrointestinal and genitourinary aberrations, limb deformations, and developmental retardation [4,5]. Life expectancy is severely limited; more than 80% of PS patients do not survive long, and according to some estimates have median survival of 2.5 days [2,6,7]. Nevertheless, only a few can survive beyond 10 years but not with serious intellectual and physical disabilities [8,9,10,11]. Early death of PS is assigned to frequent CNS and cardiopulmonary system aberrations [12].

There is no specific treatment recommended for PS. Intensive care unit level of treatment for a couple of weeks is requisite for infants. Surgery for heart defects and other abnormalities like gastrointestinal or urogenital might be needed for six-month survivors. However, CNS disorders are difficult to treat by surgery. Children surviving more than a year suffer from severe intellectual disabilities, physical abnormalities and also have a high risk of developing cancer. Most studies indicate that older women are at higher risks of delivering trisomy 13 offspring [13]. Despite the fact that there are a number of trisomy 13 cases in Saudi Arabia, no systematic study has yet been done on causative factors like maternal age, consanguinity, and parity.

PS is a multigenic complex and lethal disease of multiple congenital abnormalities associated with poor prognosis [14]. Along with CNS disorders, heart ailments, especially septal defects are leading cause of deaths [2,15]. Septal defects is a complex disorder involving hundreds of altered gene regulations and these genes are located on multiple chromosomes including chromosome 13 [16]. Chromosome 13 is 114,364,328 bp in size, representing nearly 4% of the total DNA, and encodes 308 proteins. This chromosome has 343 protein-coding genes, 622 non-coding RNA genes, and 481 pseudogenes [17].

Molecular pathway and gene ontology analysis of chromosome 13 revealed the presence of important genes like *FOXO1*, *Col4A1*, *HMGBB1*, *FLT1*, *EFNB2*, *EDNRB*, *GAS6*, *TNFSF1*, *STARD13*, *TRPC4*, *TUBA3C*, *TUBA3D*. These genes are linked with cardiovascular disorders, atrial and ventricular septal defects commonly reported in PS [18,19,20,21,22,23,24,25,26,27,28,29,30,31]. Among them, *FOXO1* is a strong transcription factor which interacts and regulates several other genes on different chromosomes, (*GATA4* (8p23.1), *GATA6* (18q11.2), *GJA1* (6q22.31), *JAG1* (20p12.2), *CITED2* (6q24.1), *RYR2* (1q43), *NKX2-5* (5q35.1), *RARA* (17q21.2), *CXCL12* (10q11.21), *SIRT1* (10q21.3), *TBX5* (12q24.21), *AKT1* (14q32.33), *CDKN2A* (9p21.3), *PCK1* (20q13.31), etc.) and are associated with septal defects in PS [32,33,34,35,36,37,38,39,40,41,42,43,44,45]. Thus, some genes like *NODAL*, *FPR1*, *AFP*, *AGO2*, *UROD*, *ZIC2* are not located on chromosome 13 but have strong association with PS.

Forkhead Box O1 (*FOXO1*) gene needs special mention. It is a member of the forkhead box O family of transcription factors located on 13q14.11. The *FOXO1* exhibits its functions by binding to promoter of downstream genes or interacting with other transcription factors [46]; both its up- or down-regulation can lead to serious consequences. It has noticeable expression in the cardiovascular system, specifically in vascular and endothelial cells, and plays a substantial role in the crucial embryonic stage [22,47]. The specific function of FOXO1 has to be determined. However, some studies strongly suggest its key role in regulation of numerous cellular functions comprising proliferation, survival, cell cycle, metabolism, muscle growth differentiation, and myoblast fusion [48,49,50]. Other observations relate it to muscle fiber-type specification highly expressed in fast twitch fiber-enriched muscles, in comparison to slow muscles. The *FOXO1* is also involved in a host of other functions: metabolism regulation, cell proliferation, oxidative stress response, immune homeostasis, pluripotency in embryonic stem cells, and apoptosis [51,52]. Besides, *FOXO1* deletion or downregulation helps to rescue heart from diabetic cardiomyopathy and increases apoptosis under stress conditions like ischemia or myocardial infarction [52,53,54,55]. The *FOXO1* is a major transcription factor in cardiac development. Thus, we see *FOXO1* null mice have underdeveloped blood vessels, whereas overexpression of the *FOXO1* gene results in reduced heart size, myocardium thickening, and eventual heart failure [18,19,20,21]. Since *FOXO1* protects cardiac tissue from a variety of stress stimuli by up-regulating anti-apoptotic, antioxidant, and autophagy genes [47,56,57], and restores metabolic equilibrium to minimize cardiac injury due to apoptosis, therefore, in PS, *FOXO1* might be a chief regulator of cardiac disorders [52]. The fact is reinforced by reports where survival is improved by suppression of upregulated *FOXO1* [18]. Given the wide range of functions of *FOXO1*, its expression rate may play a vital role in PS and we checked its inhibition via molecular docking with certain drugs.

### Molecular Docking between Candidate Drugs and FOXO1

Molecular docking, a computational simulation to screen inhibitor (ligand) compounds against biomolecule of interest, has become a crucial aspect of drug discovery approaches. Recently, repositioning or repurposing of the existing drugs is gaining attention for the treatment of diseases other than their known primary indications [58,59]. This approach could save enormous time, efforts and costs owing to the proven safety and quality of the drugs already available on the market, rather than to discover and develop novel chemical leads [60]. Similar observation on FOXO1, already implicated in a variety of functions, can specially be very promising for docking studies.

The FOXO1 protein contains 4 functional domains; (i) Forkhead domain (FKH), (ii) nuclear localization signal domain (NLS), (iii) nuclear export signal (NES), and (iv) transactivation domain (TAD). The FKH domain consists of four helices (H1–H4), two winged-loops (W1–W2), and three β strands (S1–S3), which mainly exhibits its functions as a DNA recognition and binding site. The FOXO1 regulates transcription of genes by directly binding with either 5′-GTAAA(T/C)AA-3′, or 5′-(C/A)(A/C)AAA(C/T)AA-3′ consensus sequence of downstream DNA [61,62,63]. The FOXO1 protein has thus become an extremely useful therapeutic target in many diseases including PS. Its expression can be regulated by acetylation, phosphorylation, and ubiquitination. Many potential inhibitors including leptomycin B [64], phenothiazines/trifluoperazine [65,66], bromotyrosine/psammaplysene A [67] or D4476 [68] and ETP-45658 [69], have been identified via virtual screening. Some drug candidates directly targeting FOXO1 have been patented [66]. For the docking study, we picked the FDA-approved drugs phenothiazine and its derivatives, trifluoperazine, which binds directly to the DNA binding domain of FOXO1 [70,71]. A brief introduction of both will be befitting here.

Phenothiazine (PTZ) and its derivatives are organic antihelmintic compounds presently used for important diseases like schizophrenia and bipolar disorder. Dopamine receptors are their main target. Repurposing PTZ has been tried earlier for developing novel antitumor agents [72] and Hepatitis C virus [73]. Trifluoperazine (TFP), the other derivative chosen in our studies, is a phenothiazine derivative and a dopamine antagonist, with antipsychotic and antiemetic properties. Their scaffold derivatives have also been suggested as an antiglioblastoma agent [74] and chemotherapeutic anticancer agent with high efficacy and reduced toxicity especially for oral cancer [72]. Lately, they have been shown as calmodulin antagonist [75,76].

In view of the fact that the exact mechanism is unknown as to how trisomy 13 disrupts development, heart disorders were identified as one of the most common disorders causing early death of PS patients. The present study, therefore, aims to explore the molecular interactions of 308 genes on this chromosome. We describe here the distinctive function of chromosome 13 and its key genes, especially FOXO1. We further intended to design a potential drug against FOXO1, a strong transcription factor which interacts with other key genes associated with lethal heart disorders in PS. The potential drugs to inhibit/reduce the transcriptional factor properties of FOXO1 are further explored with an aim to restore metabolic balance and limit apoptosis-induced cardiac damage.

## 2. Results

### 2.1. Cytogenetic Analysis of PS Patient

**Figure 1:**
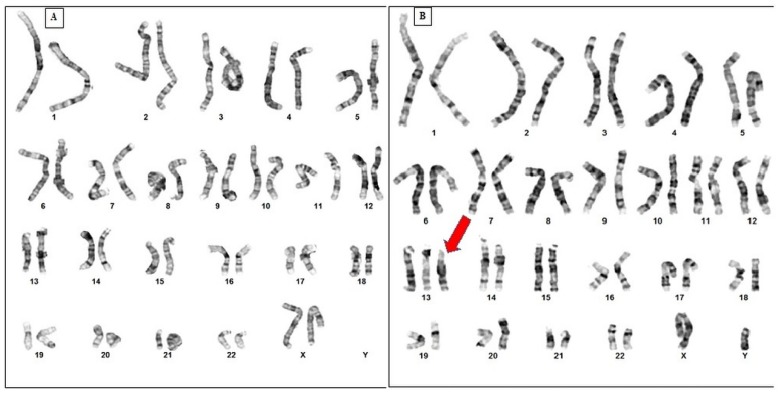
Karyotyping result; (**A**) Normal Karyotype of Healthy female and (**B**) Trisomy 13 in all cases (male = 20 and female = 17) of Patau Syndrome. Red arrow shows trisomy 13.

The prime aim of the current work was conducting genetic analysis of PS cases in the Saudi society (*n* = 37). Cytogenetic analyses were performed using G-banding technique-based karyotyping and found “full trisomy 13” in all 37 PS cases (Figure 1). The majority of individuals were newborns or children (up to 2 years), all with multiple abnormalities including heart disorders. Male to female ratio was found as 1.2:1. Analysis showed that mothers of affected individuals were above 35 years. The key clinical findings of PS observed: congenital heart defects (CHD) (61%), dysmorphic features (56%), polydactyly of hands and/or feet (53%), cryptorchidism (51%), abnormal auricles/low-set ears (47%), microphthalmia (40%), neurological disorders/microcephaly (35%), micrognathia (33%), scalp defects (31%), oral clefts (17%), microphthalmia/anophthalmia (9%), and duplication of the hallux (3%). Out of 37 cases, 31 underwent echocardiography and/or ultrasound, 21 of them showed heart defect and asymmetry of cardiac chambers. The main anatomical defects observed were arterial or ventricular septal defect, patent ductus arteriosus, pulmonic stenosis, coarctation of the aorta, tricuspid valve regurgitation, and mixed defects.

### 2.2. Molecular Pathway Analysis

**Figure 2:**
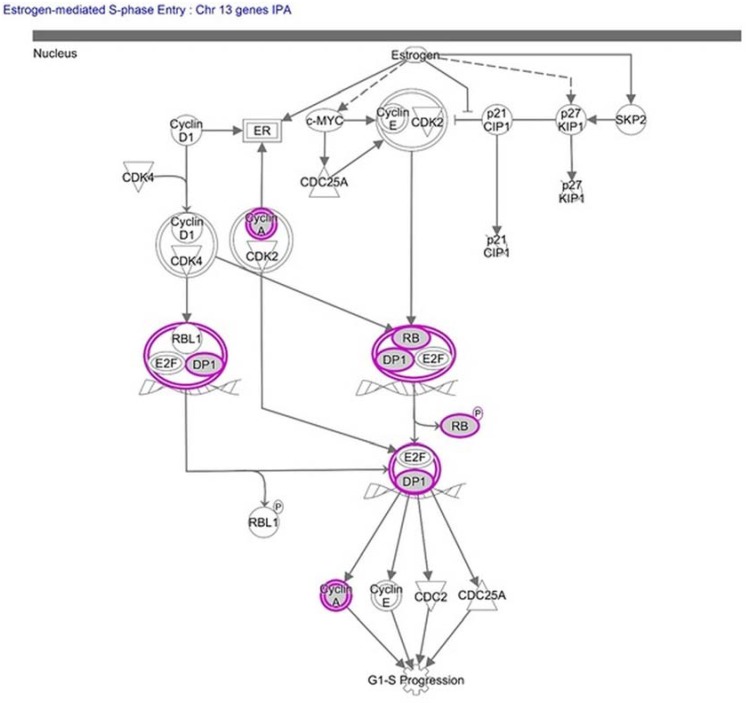
Estrogen-mediated S-phase Entry pathway derived from 308 protein coding genes of triosomy 13 (chromosome 13) using Ingenuity Pathway Analysis Tool.

Diploid status of chromosome 13 and normal expression of its genes are vital and a number of diseases are associated with its abnormalities (Table S1). However, molecular pathway and gene ontology analysis show as many as 308 protein coding genes on chr13; some of these pathogenic genes are *ATP7B*, *BRCA2*, *CAB39L*, *CKAP2*, *ESD*, *GJB2*, *GJB6*, *GPC5*, *HTR2A*, *MBNL2*, *RB1*, *SOX21*, *ZMYM2*, collectively noted for various disease associations. Other important genes such as *Col4A1*, *EFNB2*, *EDNRB*, *FLT1*, *FOXO1*, *GAS6*, *HMGB1*, *STARD13*, *TRPC4*, *TUBA3C*, *ZIC2* are specifically associated with cardiovascular disorders, atrial and ventricular septal defects—the key disorders of PS (Table 1). Ingenuity pathway analysis on 308 genes revealed canonical pathways like estrogen-mediated S-phase entry (Figure 2), gap junction signaling, cancer signaling, nitric oxide signaling in the cardiovascular system, adipogenesis pathway, VEGF signaling, cell cycle: G1/S checkpoint regulation, angiopoietin signaling, and 14-3-3-mediated signaling (Table 2). For a comprehensive idea, canonical pathways based on protein coding genes are summarized in Table 2. A cursory look shows FOXO1 to be involved in most of the canonical pathways. We focused our attention on it being strong transcription factor, interacting with and regulating many other genes on different chromosomes associated with septal defects in PS.

### 2.3. Genomic Analysis and Protein–Protein Interaction Study

**Figure 3:**
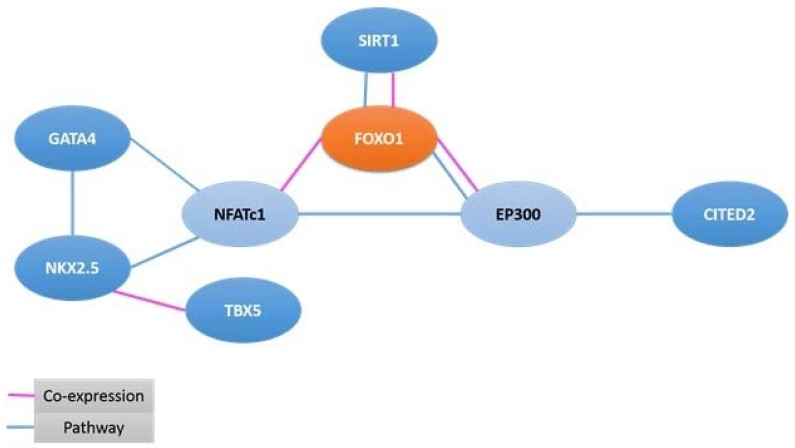
Protein–protein Interaction Partners (GATA4, NKX2-5, SIRT1, CITED, NFATc1, TBX5) of FOXO1.

The result of STRING displayed direct interaction and predicted functional relationship amid FOXO1 and its interacting proteins. The following proteins showed noticeable interactions with FOXO1: GATA4 (8p23.1), SIRT1 (10q21.3), CITED2 (6q24.1), NFATc1 (18q23), and TBX5 (12q24.21) (Figure 3). FOXO1 as transcription factor interacted with the following relevant target genes: FASLG (1q24.3), IGFBP1 (7p12.3), SOD2 (6q25.3), PPARGC1A (4p15.2), ADIPOQ (3q27.3), APOC3 (11q23.3), OSTN (3q28), BCL2L11 (2q13), CCND2 (12p13.32), and CDKN1B (12p13.1). This was predicted by text-mining application and UCSC genome browser. However, genomic analysis of PS had shown that many genes (NODAL on 10q22, FPR1 on 19q13.41, AFP on 4q13.3, AGO2 on 8q24.3, UROD on 1p34.1, ZIC2 on 13q32.3, etc.) are not directly regulated by FOXO1, rather strongly associated with PS (Table 3).

Docking using the Lamarckian Genetic Algorithm approach was employed to elucidate the basis of structural binding of PTZ and TFP to FOXO1. The result demonstrated favored binding energies ΔG in the range of −4.17 kcal/mol to −1.87 kcal/mol, respectively, with 1 molecule of PTZ showing hydrogen bond with the active site residue Ser193. Other predominant interactions for PTZ were hydrophobic (Leu163, Leu168, Val194, and Pro195) and pi–pi ring stacking non-covalent interaction with Trp189. Estimated inhibition constant, Ki values were 879.98 µM (FOXO1:PTZ) and 42.27 mM (FOXO1:TFP).

**Figure 4:**
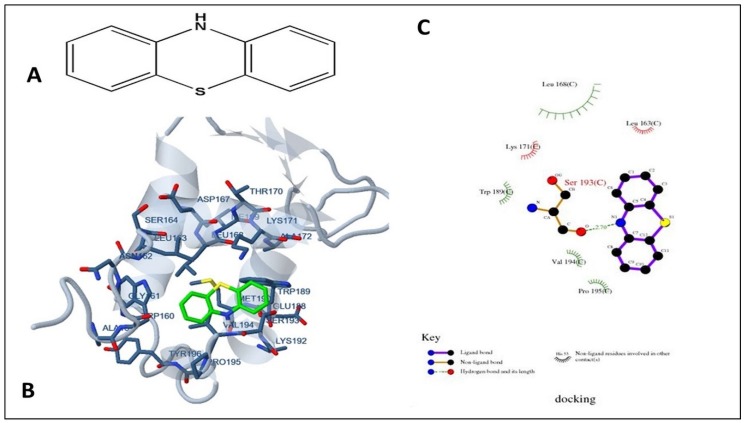
Molecular docking of phenothiazine with FOXO1 protein. (**A**) Depicting the molecular structure of phenothiazine; (**B**) Structure visualization of FOXO1 protein bound with ligand PTZ. The interacting residues are labeled in the binding site. (**C**) 2D plot of phenothiazine of FOXO1 showing ligand–protein interaction profiled by AutoDock software of Docking Server. Leu163, Leu168, Lys171, Trp189, Val194, Pro195, and Ser193 residues of FOXO1 showed crucial interactions with the phenothiazine.

**Figure 5:**
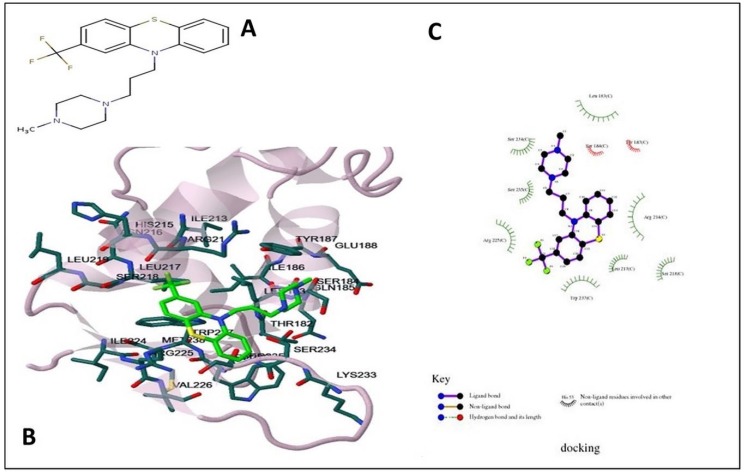
Molecular docking of trifluoperazine with FOXO1 protein. (**A**) Depicting the molecular structure of trifluoperazine; (**B**) Structure visualization of FOXO1 protein bound with ligand TFP. The binding site is shown and the interacting residues are labeled. (**C**) 2D plot of trifluoperazine of FOXO1 showing ligand–protein interaction profiled by AutoDock software of Docking Server. Leu183, Tyr187, Leu217, Arg225, Arg234, Ser184, Ser218, Ser234, Ser235 and Trp237 residues of FOXO1 showed crucial interactions with the trifluoperazine.

It was further revealed that the PTZ hydrophobic binding pocket was lined mainly with residues Leu163, Leu168, Lys171, Trp189, Val194, and Pro195, and the hydroxylic Ser193 showed crucial interactions with the ligand (Figure 4). Similarly, TFP binding site was also hydrophobic with residues Leu183, Tyr187, Leu217, Arg225, Ser234, Ser235, and Trp237. Weak interactions with TFP were seen through Ser184, Ser218, Ser234, and Ser235. Besides, non-covalent hydrogen bonding was evident between TFP’s electrophilic F1, F2, and F3 and nucleophilic O of Arg214 (Figure 5). Molecular docking analysis was done to understand the binding efficiency of the selected drugs; PTZ was found to be the better FOXO1 inhibitor as it displayed a higher negative binding energy as compared to TFP, hence, it promises to be a more effective inhibitor.

## 3. Discussion

Generally, normal development requires only two copies of autosomal chromosomes; the presence of a third copy of chromosome (trisomy) is mostly lethal to the embryo. However, trisomy 13, 18, and 21 are the only cases where development can proceed to live birth. In the present study, the age of PS patients ranged from 1 day to 2 years, the majority (*n* = 19) died within a week, 8 within a month, 9 passed a month barring 1 surviving 2 years as an exception. Studies also showed survival with trisomy 13 being miserably limited with median life expectancy of 2.5 days. The overall observation reinforces other studies where 85% of PS patients could hardly survive beyond a month [2] and rarely survive beyond 10 years [8,9,10].

It is typical to have different types of abnormalities related to chromosome 13 manifested in various disorders. Apart from PS others include 13q deletion syndrome, propionic academia, retinoblastoma, Waardenburg Syndrome, Wilson’s Syndrome, Young–Madders Syndrome and also bladder and breast cancer. In the present case, all cases had confirmed trisomy 13. However, other researchers have reported full trisomy of chromosome 13 in 70–80% of cases, mosaicism in 10–20% and translocations involving chromosomes 13 in 5–10%, besides other types of chromosomal abnormalities in 5–10% of cases [15].

The frequency of CHD in patients was 61%, which falls in the range (56 to 86%) of frequency reported by other studies [3,15,77,78]. However, relatively low frequency has been reported by Rasmussen et al. (45.7%) and Pont et al. (34.8%) [4,79].

No plausible explanation is forthcoming as to how extra genetic material (trisomy 13) causes a plethora of abnormal features like abnormal cerebral functions, a small cranium, retardation, nonfunctional eyes, and heart imperfections. We made an attempt to identify important pathogenic genes such as *EDNRB*, *ZIC2*, *ATP7B*, *GJB2*, *HTR2A* located on chromosome 13 to associate these with diseases and pathways; however, none was alone capable of the symptoms of PS.

As for exploring the pathways, it is known that the *EDNRB* gene located at 13q22.3 codes for endothelin receptor type B protein, a GPCR located on the cell surface which functions via interaction with endothelins. It activates a phosphatidylinositol-calcium, the second messenger system transmitting information from outside the cell to inside. Its highest expression is in placental tissues. Mutations in this gene have been previously linked to the congenital genetic disorder, Hirschsprung disease, alternatively called Congenital Aganglionic Megacolon. It is a neural crest development disorder characterized by absence of enteric ganglia along a variable length of the intestine causing intestinal obstruction [80]. The Zic family member 2 (*ZIC2*) gene is present at 13q32.3 genomic region and encodes a type of zinc finger protein that functions as a transcriptional repressor and regulates both early and late stages of forebrain development. Mutations in *ZIC2* gene, involving expansion of an alanine repeat at C-terminus, cause holoprosencephaly-5, a structural anomaly of the human brain [81,82,83]. It appears that a polyhistidine tract gene polymorphism is probably associated with increased risk of Holoprosencephaly. The defect appears to be due to changes in the organizer region leading to defective anterior notochord, further resulting in degradation of the prechordal plate. As a result shh signal cannot reach to the developing forebrain, vital for the formation of the two hemispheres [84]. *ZIC2* has also been linked to neural tube defects [85] and heart defects [86].

The present endeavor extended search beyond chromosome 13 and identified genes such as *NODAL*, *FPR1*, *AFP*, *AGO2*, *UROD*, *GATA4*, *GJA1*, *JAG1*, *CETED2*, *RYR2*, *NKX2-5*, *RARA*, *SIRT1*, *TBX5*, *AKT1*, and *PCK1* across genome with a view to exploring their role in PS. In doing so, two facts emerged clearly; one, the majority of PS patients had CHD and, two, all patients showed trisomy13. It thus appears that there could be a strong link between genes located on trisomy13 and heart disorders. Ingenuity pathway analysis of chr13 genes explored indicated hundreds of canonical pathways and many of them had FOXO1 as key molecules of such pathways. We applied a bioinformatics approach and searched scientific literature and identified pathogenic genes involved in CHD located on chr13 beside other chromosomes. It appears that genes of chr13 and other chromosomes might work together, either as transcription factor regulator or interacting partners. Nevertheless, *FOXO1* is a strong transcription factor activating many genes, these being: *FASLG*, *IGFBP1*, *SOD2*, *PPARGC1A*, *ADIPOQ*, *APOC3*, *OSTN*, *BCL2L11*, *CCND2*, and *CDKN1B*. The protein–protein interaction study also showed key interacting partners like GATA4, NKX2-5, SIRT1, CITED2, NFATc1 and TBX5, which are actively implicated in heart disorders and thus partly responsible for PS. 

It will not be out of place to mention GATA4, an interaction partner of FOXO1, a strong transcription factor regulating cardiac repair and remodeling. It plays an important role in cardiac development and differentiation as its abnormal expression leads to embryonic lethality [87,88,89]. Likewise, overexpression of NKX2-5 is reported as hypertrophic stimuli [90]. Interestingly, GATA4 and NKX2-5 act synergistically and regulate a myriad of cardiac genes [91,92]. Other studies showed that TBX5 is also an interaction partner of FOXO1, GATA4, and NKX2-5 and encodes transcription factors involved in the regulation of forelimb and heart development [93,94,95]. Thus, the role of GATA4, NKX2-5, and TBX5 is established in cardiogenesis; however, their role in regulating the heart septal formation is a matter of further investigation [45,96,97]. Sperling et al. are credited for reporting a direct role of *CITED2* gene mutation in CHD epigenetic factor like methylation in the promoter region of *CITED2* plays a vital role in heart disease [98,99]. The sirtuins, a family of enzymes, encoded by SIRT1–SIRT7, are highly expressed in the heart tissue and the vascular endothelium, and are pivotal regulators of lifespan and health. The SIRT1 executes its function by deacetylation of FOXO transcription factor and other key substrates; all closely linked to cardio vascular ailments. The SIRT1 inhibition is shown to be associated with septal and valvular heart defects, as well as vascular dysfunction [100,101,102].

One thing is evident clearly though multiple studies—there is a direct and indirect involvement of *FOXO1* in heart disease [52,53,54,55]. Activated *FOXO1* has a direct impact on cell survival via alteration in metabolism and turning on the cell death signaling cascade [103,104]. Overexpression of FOXO1 also causes autophagy in heart, leading to death [56,57]. A latest study had shown that knock down of *FOXO1* and *FOXO3* in the heart of Lmna^−/−^ mice results in attenuation of apoptosis with a twice increase in the survival rate [18].

A bit of inhibiting expression of FOXO1 protein will further classify its important role in regulation. It is a monomeric nuclear protein and functions primarily as transcription factors by binding to a consensus DNA sequence of promoter region of downstream genes with a DNA-binding domain, 158–248 amino acid region [63,105,106,107]. The nuclear localization of FOXO1 is tightly regulated by the post translational modifications like acetylation, methylation, phosphorylation, and ubiquitination, or simply by its interaction with proteins like 14-3-3 [105]. Studies on these lines have identified a long list of FOXO1 inhibitors to be classified into two groups: one, drugs targeting nuclear transport of FOXO1 including leptomycin B, curcumin, psammaplysene A, phenothiazines/triflouperazine, calmodulin inhibitor/calmidazolium, intracellular Ca^2+^ chelator- BAPTA-AM l; and two, drugs targeting FOXO1 signaling pathway including epigallocatechin- 3-gallate, theaflavins, hyaluronan oligosaccharides, resveratrol, apigenin, luteolin, and psammaplysene A [64,65,67].

Phenothiazines and its derivatives (trifluoperazine) are chosen from FDA-approved drugs and binds directly with DBD of FOXO1 [70,71]. The molecular docking approach was applied to determine inhibition constant, predicting binding modes and defining the specific binding sites. The results showed that both the drugs can potentially inhibit FOXO1 protein. The drug PTZ mainly interacts with hydrophobic amino acids of the DNA-binding region (158–235) of the protein target. The TFP also binds inside the DNA-binding domain but the interacting residues are different from those in the case of PTZ binding. The contact or interaction surface value of docked ligand and protein is 421.011 Å^2^ for PTZ and 612.637 Å^2^ for TFP.

The present study is mainly based on a bioinformatics approach, so it can be associated with few limitations. It is proposed to undertake in silico finding to resolve the issue, and predictions are advised to be validated before final conclusion. Our finding suggests genetic engineering potentials in future.

## 4. Materials and Methods

### 4.1. Patients

A total of 37 cases including PS, dysmorphic features, multiple congenital anomalies, CHD and cleft palate were registered from Western region of Saudi Arabia through the King Abdulaziz University Hospital, Jeddah. The majority of individuals were newborns with multiple abnormalities including heart and neurological disorders. Peripheral blood (5–10 mL) was obtained after informed consent and a complete clinical and case history was recorded. Ethical approval for the study (G/017/27) was obtained from the King Abdulaziz University clinical research ethics board dated 09-06-2009 and the study strictly followed the standard Helsinki ethical guidelines during this research work.

### 4.2. Cytogenetics Study

A standard 72 h lymphocyte culture and GTG banding (G banding by Trypsin and Giemsa) were applied to peripheral blood in all patients. Microscopic examinations were done using 50 cells for each patient. In cases of suspected mosaicism, the number expanded to one hundred cells. Chromosomes were analyzed by semi-automatic Applied Imaging Karyotyper and karyotypes were described as per the International System for Human Cytogenetic Nomenclature (ISCN, 2016) [108].

### 4.3. Molecular Pathway and Gene Ontology Analysis

Biological significance of protein coding genes of chromosome 13 was interpreted by the Ingenuity Pathways Analysis software version 338830M (Ingenuity Systems, Redwood City, CA, USA). Significance of relationships between genes and functional frameworks was indicated by Fisher’s exact test *p*-values. The percentage and number of uploaded genes/molecules matching to genes of a canonical pathway were measured for significance, expressed as a score. The Molecule Activity Predictor was employed to predict the effects of a gene/molecule on other molecules of pathway.

### 4.4. Identification of Functionally Significant Interacting Proteins of FOXO1

Search Tool for the Retrieval of Interacting Genes/Proteins (STRING version 9.1, https://string-db.org/) was used to identify significant proteins interacting with FOXO1. The biological database and web resource of known and predicted protein interactions were utilized, derived from high-throughput experimental sources, text mining and co-expression [109,110,111].

### 4.5. Molecular Docking and Drug Design

A search was made for available three-dimensional structures of FOXO1 protein in the RCSB’s PDB database and retrieved five entries: 3CO6, 3CO7, 3COA, 4LG0, and 5DUI. All these structures were DNA-bound protein complexes. We proceeded with PDB code 3CO7:C. It corresponds to UniProtKB (Q12778, https://www.uniprot.org/help/uniprotkb) and the residues 1–154 were missing from the protein chain C.

Information was collected for structure of two selected compounds; Phenothiazine and trifluoperazine from ZINC database (available online: http://zinc.docking.org). It is a database of commercially available compounds [112]. We downloaded the mol2 file for ZINC ID 00028150 and 19418959 respectively. Structural analogs of TFP (31350265 and 39546119) were not considered for the present study.

Docking calculations for predicting binding modes and energies of two ligands phenothiazine (PTZ) and trifluoperazine (TFP) to protein (FOXO1) employed DockingServer [113], and AutoDock software for gasteiger partial charges addition to the ligand atoms, combining non-polar hydrogen atoms and defining rotatable bonds. Affinity grids were generated using the Autogrid tool [114]. AutoDock parameter set- and distance-dependent dielectric functions were used in the calculation of the van der Waals and the electrostatic terms, respectively. Docking simulations were performed using Lamarckian genetic algorithm and the Solis & Wets local search algorithm (http://autodock.scripps.edu) [115]. Initial position, torsions, and orientation of the ligands were set randomly. All rotatable torsions were released during docking. All experimentation was resultant of 10 different runs set to finish after 250,000 energy evaluations. The population size was fixed to 150. Translational step of 0.2 Å, and quaternion and torsion steps of 5 were applied during the search.

### 4.6. Statistical Analysis

χ^2^ analysis and Fisher’s exact test were used to compare the clinical features and proportion of chromosomal abnormalities in PS patients. The statistical analysis was carried out using MATLAB ver R2007a (The MathWorks, Natick, MA, USA).

## 5. Conclusions

Cytogenetic analysis of 37 Saudi PS patients showed full trisomy 13 without exception. Molecular interactions study of 308 protein coding genes located on chromosome 13 led to identification of significant genes such as: *FOXO1*, *RB1*, *CCNA1*, *TFDP1*, *KL*, *IRS2*, *F10*, *F7 GJB6*, *GJA3*, *TUBA3C/TUBA3D*, *COL4A1*, *FLT1*, *KLF12*, and *ZIC2*. The pathways (Estrogen-mediated S-phase entry, Extrinsic prothrombin activation pathway, Gap junction signaling, Docosahexaenoic acid signaling, VEGF signaling, Cell cycle: G1/S checkpoint regulation, IL-3 Signaling) were explored to find an association with PS. Molecular network analysis and protein–protein interaction study indicated *FOXO1* as strong transcription factor which interacts with other key genes like *GATA4*, *CITED* and *TBX5* located on different chromosomes but associated with lethal heart disorders in PS. Lethal genetic disorders are toughest to treat and many PS newborns die within a couple of days with severe complications without proper treatment. However, patients with a less severe condition have some chance of survival and could be diagnosed with an actual problem and treated (surgery or medicine) accordingly. The in silico molecular docking studies done separately indicated phenothiazine and trifluoperazine as efficient inhibitor for FOXO1 protein as potential drugs for septal defects patients and PS. Molecular docking indicated phenothiazine to be an efficient inhibitor for FOXO1 and a candidate for future drug target, especially in septal defects patients and PS cases. It is recommended to utilize the present outcome after validation in vitro and in vivo animal model approaches.

## Figures and Tables

**Table 1:** Important pathogenic genes located on chromosome 13.

Gene Symbol	Gene Name	Cytoband	Associated Disease	Associated Pathways	Paralog
*ATP7B*	ATPase Copper Transporting Beta	13q14.3	Wilson Disease, Menkes Disease	Cardiac conduction; Ion channel transport; Transmembrane transport of small molecules	*ATP7A*
*BRCA2*	Breast cancer 2, early onset	13q13.1	Fanconi Anemia, and Breast Cancer	DNA Damage and Role of BRCA1 and BRCA2 in DNA repair	
*CAB39L*	Calcium-binding protein 39-like	13q14.2	Acute Monocytic Leukemia	RET signaling and mTOR signaling pathway	*CAB39*
*COL4A1*	Collagen Type IV Alpha 1 Chain	13q34	Coronary artery disease	Collagen chain trimerization, Integrin Pathway, ERK Signaling.	*COL4A5*
*DZIP1*	DAZ interacting zinc finger protein 1	13q32.1	Acrodermatitis Enteropathica, Zinc-Deficiency Type	Hedgehog signaling and GPCR signaling.	*DZIP1L*
*EDNRB*	Endothelin receptor type B	13q22.3	Waardenburg Syndrome	Calcium signaling pathway and Prostaglandin Synthesis and Regulation	*EDNRA*
*ESD*	S-formylglutathione hydrolase	13q14.2	Wilson Disease and Leukocoria	Glutathione metabolism	
*FOXO1*	Forkhead box O 1	13q14.11	Rhabdomyosarcoma 2, Alveolar and Rhabdomyosarcoma	RET signaling; PI3K/AKT activation; Common Cytokine Receptor Gamma-Chain Family Signaling Pathways; AGE/RAGE pathway	*FOXO3*
*FLT1*	Fms-related tyrosine kinase 1	13q12.3	Anal Canal Squamous Cell Carcinoma and Eclampsia	p70S6K Signaling and Focal Adhesion	*KDR*
*GAS6*	Growth Arrest Specific 6	13q34	Sticky platelet Syndrome, Acute Maxillary Sinusitis, Mesangial Proliferative Glomerulonephritis	Apoptotic Pathways in Synovial Fibroblasts, GPCR Pathway, ERK Signaling	*PROS1*
*GJB2*	Gap junction protein, beta 2, 26 kDa (connexin 26)	13q12.11	Vohwinkel Syndrome and Bart–Pumphrey Syndrome	Development Slit-Robo signaling and Gap junction trafficking.	*GJB6.*
*GJB6*	Gap junction protein, beta 6 (connexin 30)	13q12.11	Ectodermal Dysplasia 2, Clouston Type and Deafness, Autosomal Dominant 3B	Gap junction trafficking; Vesicle-mediated transport	*GJB2*
*GPC5*	Glypican-5	13q31.3	Simpson–Golabi–Behmel Syndrome and Tetralogy of Fallot	Glycosaminoglycan metabolism	*GPC3*
*HMGB1*	Box 5 Box 1	13q12.3	13q12.3 Microdeletion Syndrome, Adenosquamous Gallbladder Carcinoma	Activated TLR4 signaling; Cytosolic sensors of pathogen-associated DNA; Innate Immune System	*HMGB2*
*HTR2A*	5-HT2A receptor	13q14.2	Schizophrenia; Major Depressive Disorder	Calcium signaling pathway; Signaling by GPCR	*HTR2C*
*MIPEP*	Mitochondrial intermediate peptidase	13q12.12	Combined Oxidative Phosphorylation Deficiency 31		
*PCCA*	Propionyl Coenzyme A carboxylase, alpha polypeptide	13q32.3	Propionicacidemia and PCCA-Related Propionic Acidemia.	Metabolism and HIV Life Cycle.	*MCCC1*
*RB1*	Retinoblastoma 1	13q14.2	Retinoblastoma and Small-Cell Cancer of the Lung, Somatic.	Arrhythmogenic right ventricular cardiomyopathy (ARVC) and DNA Damage	*RBL2*
*RCBTB1*	RCC1 and BTB domain-containing protein 1	13q14.2	Retinal Dystrophy with Or Without Extraocular Anomalies.		*RCBTB2*
*RGCC*	Regulator of cell cycle RGCC	13q14.11	Renal Fibrosis and Retinal Cancer	TP53 Regulates Transcription of Cell Cycle Genes	
*RNR1*	Encoding RNA, ribosomal 45S cluster 1	13p12	Idiopathic Bilateral Vestibulopathy and Congenital Cytomegalovirus	Viral mRNA Translation	
*SLITRK6*	SLIT and NTRK-like protein 6	13q31.1	Deafness and Yopia and Autosomal Recessive Non-Syndromic Sensorineural Deafness		*SLITRK5*
*SOX21*	Transcription factor SOX-21	13q32.1	Mesodermal Commitment Pathway and ERK Signaling.	Mesodermal Commitment Pathway; ERK Signaling	*SOX14*
*STARD13*	StAR-Related Lipid Transfer Domain Containing 13	13q13	Hepatocellular Carcinoma, Arteriovenous Malformations of the Brain, Fibrosarcoma of Bone	p75 NTR receptor-mediated signaling, Signaling by GPCR, Signaling by Rho GTPases	*STARD8*
*TPT1*	Translationally controlled tumor protein (TCTP)	13q14.13	Urticaria and Asthma	DNA Damage and Cytoskeletal Signaling	
*TRPC4*	Transient Receptor Potential Cation Channel Subfamily C Member 4	13q13.3	Photosensitive Epilepsy	Developmental Biology, Ion channel transport, Netrin-1 signaling	*TRPC5*
*TSC22D1*	TSC22 domain family protein 1	13q14.11	Salivary Gland Cancer and Brain Sarcoma	Development TGF-beta receptor signaling and Ectoderm Differentiation	*TSC22D2*
*TUBA3C*	Tubulin Alpha 3C	13q12.11	Clouston Syndrome, nonsyndromic Deafness, Kabuki Syndrome 1	Development Slit-Robo signaling, Cooperation of Prefoldin and TriC/CCT in actin and tubulin folding	*TUNA3D*
*XPO4*	Exportin-4	13q12.11	Conjunctival Degeneration and Pinguecula	eIF5A regulation in response to inhibition of the nuclear export system and Ran Pathway	
*ZIC2*	Zic Family Member 2	13q32.3	Holoprosencephaly 5 and Zic2-Related Holoprosencephaly	Mesodermal Commitment Pathway	*ZIC1*
*ZMYM2*	Zinc finger MYM-type protein 2	13q12.11	Lymphoblastic Lymphoma and 8P11 Myeloproliferative Syndrome	HIV Life Cycle and FGFR1 mutant receptor activation	*ZMYM3*

**Table 2:** Top canonical pathways determined by Ingenuity pathway analysis tools based on protein coding genes located on chromosome 13.

Canonical Pathways	−log (*p* Value)	Ratio	Molecules
Estrogen-mediated S-phase Entry	2.06	0.115	RB1, CCNA1, TFDP1
Cancer Signaling	1.69	0.052	RB1, FOXO1, TFDP1, KL, IRS2, CDK8, SMAD9, TFDP1, ARHGEF7
Extrinsic Prothrombin Activation Pathway	1.56	0.125	F10, F7
Role of p14/p19ARF in Tumor Suppression	1.5	0.071	RB1, KL, IRS2
Gap Junction Signaling	1.41	0.036	GJB6, KL, GJA3, TUBA3C/TUBA3D, IRS2, GJB2, HTR2A
Docosahexaenoic Acid (DHA) Signaling	1.27	0.057	FOXO1, KL, IRS2
Aldosterone Signaling in Epithelial Cells	1.24	0.035	SACS, KL, HSPH1, DNAJC3, IRS2, DNAJC15
FGF Signaling	1.2	0.044	KL, FGF9, FGF14, IRS2
GP6 Signaling Pathway	1.18	0.038	COL4A1, KL, IRS2, COL4A2, KLF12
Adipogenesis pathway	1.17	0.037	RB1, SAP18, SMAD9, FOXO1, KLF5
VEGF Signaling	1.08	0.040	FOXO1, FLT1, KL, IRS2
Cell Cycle: G1/S Checkpoint Regulation	1.04	0.046	RB1, FOXO1, TFDP1
ErbB2-ErbB3 Signaling	0.994	0.044	FOXO1, KL, IRS2
Nitric Oxide Signaling in the Cardiovascular System	0.988	0.037	FLT1, KL, SLC7A1, IRS2
Coagulation System	0.948	0.057	F10, F7
Angiopoietin Signaling	0.875	0.039	FOXO1, KL, IRS2
Role of NANOG in Mammalian Embryonic Stem Cell Pluripotency	0.866	0.0333	SMAD9, KL, CDX2, IRS2
IL-3 Signaling	0.805	0.036	FOXO1, KL, IRS2
Actin Cytoskeleton Signaling	0.801	0.027	KL, FGF9, DIAPH3, ARHGEF7, FGF14, IRS2
14-3-3-mediated Signaling	0.778	0.030	FOXO1, KL, TUBA3C/TUBA3D, IRS2
IL-7 Signaling Pathway	0.774	0.034	FOXO1, KL, IRS2
HMGB1 Signaling	0.77	0.030	HMGB1, KL, IL17D, IRS2
NF-κB Signaling	0.769	0.028	TNFSF11, FLT1, KL, IRS2, TNFSF13B

**Table 3:** Key genes strongly associated with the survival of PS patient.

Gene Symbol	Gene Name	Cytoband	Associated Disease	Associated Pathways	Paralog
NODAL	Nodal Growth Differentiation Factor	10q22	Visceral Heterotaxy 5 (HTX5) and Nodal-Related Visceral Heterotaxy	Mesodermal Commitment Pathway and Signaling pathways regulating pluripotency of stem cells	GDF3
FPR1	Formyl Peptide Receptor 1	19q13.41	Susceptibility to Localized Juvenile Periodontitis and Periodontitis 1, Juvenile	Signaling by GPCR and Peptide ligand-binding receptors	FPR2
AFP	Alpha Fetoprotein	4q13.3	Alpha-Fetoprotein Deficiency and Hereditary Persistence of Alpha-Fetoprotein	Glucocorticoid receptor regulatory network and Embryonic and Induced Pluripotent Stem Cell Differentiation Pathways and Lineage-specific Markers	ALB
AGO2	Argonaute RISC Catalytic Component 2	8q24.3	Chromosome 18P Deletion Syndrome and Gum Cancer	RET signaling and Translational Control.	AGO1
UROD	Uroporphyrinogen Decarboxylase	1p34.1	Porphyria Cutanea Tarda and Urod-Related Porphyrias	Metabolism and Porphyrin and chlorophyll metabolism	
GATA4	GATA Binding Protein 4	8p23.1	Testicular Anomalies with or without Congenital Heart Disease and Atrial Septal Defect 2	Response to elevated platelet cytosolic Ca2+ and Human Embryonic Stem Cell Pluripotency	GATA6
GATA6	GATA Binding Protein 6	18q11.2	Pancreatic Agenesis and Congenital Heart Defects and Atrioventricular Septal Defect 5	Mesodermal Commitment Pathway and Response to elevated platelet cytosolic Ca2+	GATA4
GJA1	Gap Junction Protein Alpha 1	6q22.31	Oculodentodigital Dysplasia and Syndactyly, Type Iii	Development Slit-Robo signaling and Arrhythmogenic right ventricular cardiomyopathy	GJA3
JAG1	Jagged 1	20p12.2	Alagille Syndrome 1 and Tetralogy of Fallot	Signaling by NOTCH1 and NOTCH2 Activation and Transmission of Signal to the Nucleus	JAG2
CITED2	Cbp/P300 Interacting Transactivator with Glu/Asp Rich Carboxy-Terminal Domain2	6q24.1	Atrial Septal Defect 8 and Ventricular Septal Defect 2	Cellular Senescence (REACTOME) and Transcriptional regulation by the AP-2 (TFAP2) family of transcription factors	CITED1
RYR2	Ryanodine Receptor 2	1q43	Ventricular Tachycardia, Catecholaminergic Polymorphic, 1 and Arrhythmogenic Right Ventricular Dysplasia 2	Calcium signaling pathway and Arrhythmogenic right ventricular cardiomyopathy	RYR3
NKX2-5	NK2 Homeobox 5	5q35.1	Atrial Septal Defect 7, With or Without Av Conduction Defects and Tetralogy of Fallot	Human Embryonic Stem Cell Pluripotency and NFAT and Cardiac Hypertrophy	NKX2-3
RARA	Retinoic Acid Receptor Alpha	17q21.2	Leukemia, Acute Promyelocytic, Somatic and Myeloid Leukemia	Nuclear Receptors in Lipid Metabolism and Toxicity and Activated PKN1 stimulates transcription of AR (androgen receptor) regulated genes KLK2 and KLK3.	RARB
CXCL12	C-X-C Motif Chemokine Ligand 12	10q11.21	HIV-1 and AIDS Dementia Complex	p70S6K Signaling and Akt Signaling	
SIRT1	Sirtuin 1	10q21.3	Xeroderma Pigmentosum, Group D and Ovarian Endodermal Sinus Tumor	Longevity regulating pathway and E2F transcription factor network	SIRT4
TBX5	T-Box 5	12q24.21	Holt–Oram Syndrome and Aortic Valve Disease 2	Human Embryonic Stem Cell Pluripotency and Cardiac conduction.	TBX4
AKT1	AKT Serine/Threonine Kinase 1	14q32.33	Cowden Syndrome 6 and Proteus Syndrome, Somatic	Transcription Androgen Receptor nuclear signaling and E-cadherin signaling in keratinocytes	AKT3
CDKN2A	Cyclin Dependent Kinase Inhibitor 2A	9p21.3	Pancreatic Cancer/Melanoma Syndrome and Melanoma and Neural System Tumor Syndrome	DNA Damage and Bladder cancer	CDKN2B
PCK1	Phosphoenolpyruvate Carboxykinase 1	20q13.31	Pepck 1 Deficiency and Phosphoenolpyruvate Carboxykinase-1, Cytosolic, Deficiency	Abacavir transport and metabolism and Citrate cycle (TCA cycle)	PCK2

## Supplementary Materials

Supplementary materials can be found at http://www.mdpi.com/1422-0067/19/11/3547/s1.
