# Supplementary material for: Role of Overexpressed Transcription Factor FOXO1 in Fatal Cardiovascular Septal Defects in Patau Syndrome: Molecular and Therapeutic Strategies
Source: Int J Mol Sci. 2018 Nov 10;19(11):3547. doi: 10.3390/ijms19113547 (PMC6274780; doi:10.3390/ijms19113547)
Supplement: Supplementary file 1 [file ijms-19-03547-s001.pdf]

Table S1. Diseases and disorders associated with structural and/or molecular abnormalities of chromosome 13.

**Disease** *	**Description**
Patau syndrome or Trisomy 13	It is a chromosomal anomaly caused by the presence of an extra chromosome 13 and is characterized by severe facial dysmorphism, ocular anomalies, postaxial polydactyly and severe psychomotor retardation. However, brain malformations (holoprosencephaly), visceral malformations (cardiopathy) and heart septal defect are lethal.
13q deletion syndrome	Deletion of long arm (q) of chromosome 13. The severity of the condition and symptoms (developmental delay, intellectual disability, behavioral problems and distinctive facial features) depend on the size and location of the deleted genetic material (genes).
Propionic acidemia	Organic acid disorder is caused by an abnormal formation and accumulation of particular organic acids that leads to poor feeding, vomiting, loss of appetite, weak muscle tone (hypotonia), lack of energy (lethargy), heart abnormalities, seizures, coma, and even death.
Retinoblastoma	It is rare type of eye (retina) cancer in children and caused by deletions of RB1 gene in 13q14 region. Some children may also have mental retardation, slow growth, and abnormal facial features.
Waardenburg syndrome	It is a multigene congenital disorder involving deletion of 2q36.1 (PAX3), 3p13 (MITF), 13q22.3 (EDNRB), and 20q13 (EDN3). Major symptoms are hearing loss and pigmentary abnormalities of the hair, skin, and eyes.
Wilson's disease	Wilson's disease is a result of malfunction of ATP7B gene of chr13 that leads to accumulation of copper in liver and cause hepatic, kidney and neurological disease.
Young–Madders syndrome or Pseudo-trisomy 13 syndrome	It is a genetic disorder with undetermined defects of chr13 but very likely resulting from abnormal 13q32.3 (ZIC2) and symptoms are similar to trisomy 13 syndrome like holoprosencephaly, polydactyly, facial malformations and mental retardation.

* Bladder cancer, Breast cancer, Heterochromia, Schizophrenia Hirschsprung's disease, Maturity onset diabetes of the young type 4, Nonsyndromic deafness, Chronic Lymphocytic Leukemia (Acquired defect) are other important diseases and disorders associated with abnormalities in chromosome 13.
